# *In silico* evaluation of the influence of the translocon on partitioning of membrane segments

**DOI:** 10.1186/1471-2105-15-156

**Published:** 2014-05-21

**Authors:** Dominique Tessier, Sami Laroum, Béatrice Duval, Emma M Rath, W Bret Church, Jin-Kao Hao

**Affiliations:** 1 INRA, UR1268 Biopolymères Interactions et Assemblages, Nantes F-44316, France; 2 LERIA, 2 Boulevard Lavoisier, Angers 49045, France; 3 Group in Biomolecular Structure and Informatics, Faculty of Pharmacy, The University of Sydney, Sydney, NSW 2006, Australia

**Keywords:** Translocon, Transmembrane helix prediction, Local search algorithm

## Abstract

**Background:**

The locations of the TM segments inside the membrane proteins are the consequence of a cascade of several events: the localizing of the nascent chain to the membrane, its insertion through the translocon, and the conformation adopted to reach its stable state inside the lipid bilayer. Even though the hydrophobic h-region of signal peptides and a typical TM segment are both composed of mostly hydrophobic side chains, the translocon has the ability to determine whether a given segment is to be inserted into the membrane. Our goal is to acquire robust biological insights into the influence of the translocon on membrane insertion of helices, obtained from the *in silico* discrimination between signal peptides and transmembrane segments of bitopic proteins. Therefore, by exploiting this subtle difference, we produce an optimized scale that evaluates the tendency of each amino acid to form sequences destined for membrane insertion by the translocon.

**Results:**

The learning phase of our approach is conducted on carefully chosen data and easily converges on an optimal solution called the PMIscale (Potential Membrane Insertion scale). Our study leads to two striking results. Firstly, with a very simple sliding-window prediction method, PMIscale enables an efficient discrimination between signal peptides and signal anchors. Secondly, PMIscale is also able to identify TM segments and to localize them within protein sequences.

**Conclusions:**

Despite its simplicity, the localization method based on PMIscale nearly attains the highest level of TM topography prediction accuracy as the current state-of-the-art prediction methods. These observations confirm the prominent role of the translocon in the localization of TM segments and suggest several biological hypotheses about the physical properties of the translocon.

## Background

The proteins transported into the endoplasmic reticulum (ER) include transmembrane (TM) proteins which become embedded in the ER membrane, and water-soluble proteins which are fully translocated across the ER membrane and released into the ER lumen. Proteins are guided to the ER while they are synthesized on the ribosome by a protein complex – the Signal Recognition Particle – that recognizes a targeting signal localized in the growing polypeptide. The targeting signals are either N-terminal signal sequences called signal peptides (SP) or, in the case of many membrane proteins that lack signal peptides, the first TM segment which is called a signal anchor. Insertion into the ER is then mediated by an evolutionarily conserved membrane protein complex, the translocon. This protein conduction channel provides a passage for proteins across the membrane as well as a means to integrate nascent proteins into the membrane through a lateral exit gate. When this gate is opened, TM segments may move from the aqueous interior of the channel into the lipid phase of the membrane. Finally, the stably folded membrane protein raises a minimum free energy inside the lipid bilayer. A large number of computational methods are available for detecting signal peptides (SignalP [1], Signal-3L [2], Signal-CF [3], PrediSi [4]) or localizing TM segments (TMHMM [5], Phobius [6], MemBrain [7]). Complete reviews of the advantages and shortcomings of these methods are available in [8,9]. Only a few of them, such iPSORT [10] or SOMRuler [11] concentrate on making the relationships between amino acid sequences and signal peptides or TMH transparent.

The objective of our study is to evaluate the influence of the translocon on the partitioning of membrane segments. In 2005, Hessa et al. attempted to elucidate this phenomenon of selective helical TM segment movement through the ER translocon site with a series of experiments. They developed an experimental system that makes it possible to measure the membrane insertion efficiency of a large set of hydrophobic model segments [12,13]. These studies suggested that insertion or not of a helical TM segment is fundamentally a problem of equilibrium thermodynamics for most of the TM segments. According to this hypothesis, the membrane insertion of a TM segment mainly depends on the local contribution of each amino acid inside the translocon channel. Moreover, it also suggested a strong position-dependence within the hydrophobic segment for each of the 20 amino acids. Nevertheless, this approach which leads to the so-called hydrophobicity biological scale (BH) is uniquely based on the variation of an engineered TM segment included in the protein leader peptidase (Lep).

In order to benefit from the BH scale, two translocon-based prediction tools have been developed to predict the localization of TM segments: ΔG predictor [13] and SCAMPI [14]. These tools are based on the same calculation of the free energy cost of insertion but they use different algorithms. Moreover, whereas SCAMPI calculates the energy of peptides with a fixed length of 21 amino acids, ΔG predictor allows length corrections. Even though prediction accuracies obtained by ΔG predictor or SCAMPI are outperformed by tools such as OCTOPUS [15] or Philius [16], such methods are however extremely useful as they attempt to give clues to explain biological observations and help us to more precisely understand the mechanism governing the insertion of helical TM. In the future, an accurate prediction could identify segments that are borderline in their classification, and that are therefore able to switch between TM and non-TM configuration states. Such switches are known to occur in a number of cases but it is difficult to evaluate their prevalence at this time [17]. Finally, a detailed understanding of topology determinants can lead to the design of hydrophobic helices with biomedical applications.

In their work, Hessa et al. do not focus on the problematic aspects of signal peptides and unfortunately, tools developed from their results have difficulty in differentiating them from TM segments. Nevertheless, even though the central region of signal peptides – the h-region - and a typical TM segment are both composed of mostly hydrophobic side chains, the translocon has the ability to sort them. If the translocon can determine whether or not a given segment should be inserted into the membrane, we can expect that essential elements promoting the phenomenon could be captured by *in silico* exploitation of the difference between the amino acid composition of the hydrophobic core of signal peptides and TM segments. Such an approach could benefit from a large number of learning datasets but these data must be chosen carefully. Although the UniprotKB/SwissProt annotations cannot be regarded as experimentally established topography data, we chose this databank to construct our training data set because it allows the selection of only eukaryotic proteins with a type II or type III signal anchor annotation. To decrease the bias introduced by the use of the TM prediction tools which may be the origin of annotations in UniprotKB/SwissProt, we considered that TM segments are actually not precisely located. Several studies have shown that some TM segments in polytopic proteins need to cooperate during the membrane insertion step [18]. The exclusion of polytopic proteins from the training data eliminates TM segments that depend on other parts of the protein for efficient insertion and folding. We insist on this restriction because the inclusion of polytopic proteins in training data may compromise the prediction accuracy of bitopic proteins and vice-versa.

The *in silico* elaboration of a new scale can be considered as an optimization problem and a local search approach is an effective technique to solve it. In previous work [19,20], our scale assigned a symmetric curve to each amino acid in order to take into account its position inside the translocon, but we only partially succeeded in obtaining stable curves. In this study, we instead assign an average value for each amino acid and arrive at the striking observation that, with very few parameters, this new scale, called PMIscale, obtains quite good results, both for discriminating SP from TM segments and for capturing a large part of the information required to locate TM segments along the membrane proteins.

## Results and discussion

### The new PMIscale

The local search algorithm used to find a new scale that discriminates signal peptides from signal anchors quickly converges on an optimal solution that results in the PMIscale displayed in Table 1. A high value corresponds to a high preference for TM segment insertion.

**Table 1:** Amino acid PMIscale values

A	1.8	G	1.6	M	1.9	S	-1.8
C	2.1	H	-0.2	N	-3.5	T	2.3
D	-8.5	I	6.5	P	-4.6	V	4.2
E	-11.5	K	0.1	Q	-4.5	W	5.7
F	5.8	L	3.8	R	0.5	Y	6.1

When compared with other hydrophobicity scales, we observe that the PMIscale highlights that more efficient promotion into the membrane insertion occurs for the aromatic side chains Trp, Tyr, and Phe. These results suggest that these amino acids participate strongly in the recognition of TM helices by the translocon. In addition, PMIscale does not penalize basic amino acids – Arg, His, Lys - as much as the other scales even though the lipid bilayer does not favor their presence, suggesting that the translocon may also play a major role in the insertion of these amino acids. This result agrees with the computer simulations of a helix containing an arginine sidechain conducted specifically to consider the sidechain moving from the translocon to the lipid bilayer [21].

### Evaluation of the discrimination between SP and TM

As shown in Table 2, the PMIscale enables a significantly improved discrimination between SP and TM than other scales when a sliding-window approach is used as described in the Methods section. The quality of such a classification system can be evaluated by the Area Under the ROC curve (AUC) [22]. The performance of PMIscale for discriminating SP from signal anchors is excellent for the SWPTest dataset (AUC = 0.932) and, unlike other scales, it also exhibits suitability for the task of discriminating SP from TM segments as shown by our benchmark for the PDBTMSeg dataset (AUC = 0.803). For information purposes, Additional file 1: Table S1 also provides insights into the effectiveness of PMIscale compared with two widely used machine learning-based methods [1,6].

**Table 2:** The AUC quality assessment of the discrimination between SP and TM segments on several datasets

	***SWPTest***	***ScampiHigh***	***ScampiLow***	***PDBTMSeg***
PMIscale	**0.932**	**0.86**	**0.845**	**0.803**
K&D	0.829	0.662	0.691	0.636
GES	0.793	0.736	0.737	0.667
BH-2005	0.895	0.752	0.733	0.676
TM tendency	0.887	0.792	0.814	0.756
AvgH	0.837	0.706	0.726	0.67

The scales shown are from the following references: KD [23]; GES [24]; BH [12]; TM tendency [25]; AvgH [26].

### Prediction of membrane proteins in proteome-wide studies

If PMIscale conveys relevant information about the translocon mechanisms, it should also be able to predict accurately whether a protein is a membrane protein or not. It was previously observed in [27] that the energy required for the insertion of the TM segment of a bitopic protein must be higher than the energy required by the insertion of the following TM segments. In our approach, we extended this observation with the notion of ‘first TM segment’ – the TM segment of a bitopic protein or the first TM segment of a polytopic protein - and we introduced two thresholds in the TM localization algorithm: τfirst for the insertion of the first TM segment deduced from the threshold that separates signal peptides from signal anchors, and τnext for the following TM segments. In addition it was also observed that *in vitro*, the SRP binding to the ribosome nascent chain declines when the nascent chain reaches a length of 110-140 amino acids [28]. Therefore when evaluating methods based on a sliding-window approach, we added the constraint that the signal anchor is not located after that limit. Consequently τfirst determines if a protein is a membrane protein or not for only that limited N-terminal part of the protein.

A guideline to proteome-wide α-helical membrane protein topology has been published recently [29] giving the opportunity to compare the PMIscale predictions with 18 algorithms on control datasets. We compared PMIscale on two benchmark datasets extracted from this work that permit evaluation of membrane-inserted proteins. We also performed a comparison with the ΔG predictor method, because this method is directly based on the Hessa et al. [12] biological scale. The first dataset is composed of cytosolic proteins without any signal peptide or TM segment. For this dataset, the PMIscale based predictor predicts 2.8% proteins with at least one TM segment which places it as one of the three best methods, 12% better than the average performance of the evaluated programs in [29]. The second dataset is composed of extracellular proteins that contain a signal peptide but no TM segment. The PMIscale predicts 10.2% proteins with at least one TM segment, which is 30% higher and therefore significantly better than the average performance of the 18 more sophisticated methods. We can note that over-prediction errors are much less abundant with PMIscale than when other hydropathy plot methods are used, placing it at the same level as the best methods Phobius [30], Phillius [16] and Polyphobius [31]. The ΔG predictor is not adapted to this situation (it predicts 70% of proteins as having at least one TM segment), which indicates that the BH scale may not differentiate signal peptides from TM segments.

The last benchmark dataset we tested was extracted from the benchmark server developed by Rath et al. [32] which offers general and specialized assessment of existing and novel membrane helix prediction methods. In this dataset, the SP was cleaved from the mature protein. We evaluated the standard benchmark referred to as the TMH_1/2MH_OPM_BB_SOLB dataset (which consists of sequences having less than 30% similarity to each other possessing membrane helices that are long enough to traverse the membrane and known membrane helices that do not traverse the entire membrane). PMIscale achieves a good performance that is 21% better than the average of the 53 available methods. Performance result details are available in Additional file 1: Table S2.

### Prediction of TM localization in membrane proteins

The initial objective of PMIscale was to provide information about the translocon passage. However, our experiments demonstrated that this scale is also able to localize TM segments in protein sequences. To evaluate this point, we used two benchmark datasets. One is composed of 1311 G-protein coupled receptors (GPCRs) extracted from [29]. The particularity of this data set is that the topology of GPCRs is challenging to predict, as several of the TM helices are uncharacteristically hydrophilic. In our benchmark, the prediction is regarded as correct if it contains all 7 and only 7 TM segments. In this case, the PMIscale prediction is lower than the average of the evaluated methods with only 37% of the proteins predicted with 7 TM segments. This is not surprising because TM helices in the case of the GPCRs could probably not be predicted with any method limited to the composition of the individual TM segments alone. The usefulness of high accuracy prediction of transmembrane inter-helix contacts has been demonstrated in this particular protein family [33]. In this more challenging case, sophisticated methods command an advantage because they additionally extract information from global features of the sequences, rather than using only local features of the TM segments. Additional global information about the positively charged residues of the alternate sides of the membrane and the general bias of the charges between regions of the proteins has also been proven to be useful [34]. Moreover, with this particular family, the prediction power can be improved by multiple sequence alignment information. We also note that prediction performances on this particular dataset vary a lot between algorithms.

The second dataset was a standard benchmark data subset suggested by [32], that we used to compare our novel PMIscale to 52 transmembrane helix prediction methods freely available to be run in batch mode. The evaluation was limited to topography scores, i.e. the accuracy per protein sequence and the accuracy per segment. The results of specificity and the percentage of correctly predicted proteins show that the performance of PMIscale is significantly better than average. However, PMIscale’s performance for sensitivity is slightly lower than the average of the methods. The results are summarised in Table 3, and a detailed comparison with each method is available as supplementary data in Additional file 1: Table S2. We also used this dataset to evaluate the PMIscale-based predictions when the length of the sliding window is modified and we observe a moderate degradation of the performance when the window is set to 21 or 25 amino acids (shown in Additional file 1: Table S3). Finally, we measured the impact on prediction accuracy when the values of the thresholds τfirst and τnext are modified. The results show that higher values improve the specificity of predictions whereas lower values are able to identify all the membrane proteins with very few exceptions. Performance results for modifications in thresholds are available in Additional file 1: Table S4.

**Table 3:** Benchmark measures on a dataset extracted from the Rath et al. benchmark web server

	**Sensitivity (%)**	**Specificity (%)**	**Correctly predicted sequences (%)**
PMIscale	80	90	88
Averaged performance of 52 methods	82.6	70.7	66.2

This benchmark contains 599 sequences – 133 membrane proteins TMH or β-barrel and 466 soluble proteins – including 483 membrane helices; the averaged performance was calculated on the 52 transmembrane helix prediction methods with topography information available on the web server - TMLOOP method is not taken into account.

## Conclusions

PMIscale is able to distinguish signal peptides from TM segments as the translocon does. Moreover, accuracies obtained by the PMIscale on all the benchmark datasets are close to those of the most accurate and sophisticated methods. This occurs despite the fact that our method is based on a simple algorithm and has only 22 parameters - 20 values from the PMIscale and 2 thresholds, τfirst and τnext. Information used in the predictions here is strictly limited to the amino acid composition of the protein segment and is derived from the bias observed between the composition of the signal peptides and the signal-anchor segments. Compared to usual sliding-window approaches used to precisely localize where the TM segments are, improvements in predictions are due to the new scale, and also due to the introduction of a threshold term which differentiates the first segment.

Our *in silico* results are consistent with the experimental results of Hessa et al., as they suggest that the translocon passage is the major factor that influences the TM segment positions. Nevertheless, we can also note that taking into account the position of the amino acids inside the translocon does not give rise to much predictive benefit in the comparison of the performances of the SCAMPI, ΔG predictor and PMIscale methods.

Some particular protein families such as the GPCRs require more specific algorithms for the precise localization of their TM segments. However, PMIscale could be very helpful for proteome-scale or genome-scale studies: the PMIscale-based sliding-window predictor is easy to use, quick and efficient which is important for large-scale genome processing. Moreover, if the objective of a prediction is to elaborate target lists that either exclude or specifically select integral membrane proteins as it is sometimes required in structural genomics projects, it is easy to modify the thresholds τfirst and τnext to adjust the resulting inclusivity levels. An online service for individual predictions and a stand-alone PMIscale package for genome-scaled predictions written in Perl are provided on the web site http://wwwappli.nantes.inra.fr/bioinfoweb.

## Methods

### Selection of the data sets

The major problem with the training data sets of TM prediction methods is the small number of membrane proteins in the PDB database [35]. This proportion is less than 2% according to the PDBTM database [36]. It has also been shown that the commonly chosen test sets are biased and, consequently, the reliability of the predictors could be lower than reported [37]. Even though the UniprotKB/SwissProt annotations cannot be regarded as experimentally established topography data, we chose this database and retrieval tools [38] to construct our training dataset because it allows the selection of exclusively eukaryotic proteins with a signal anchor annotation. A signal anchor serves the purpose of the ER targeting as does a signal peptide, but it inserts into the membrane while the signal peptide does not. For our purpose, we selected reviewed eukaryotic proteins marked with a “Signal-anchor for type II membrane protein” or “Signal-anchor for type III membrane protein” annotation [6]. We added the 10 adjacent amino acids to the TM segment or fewer if the number of adjacent amino acids was lower than 10. CD-HIT [39] was then used to obtain a final non-redundant protein set with an identity cut-off at 30%.

The signal peptide dataset was extracted from SwissProt with only eukaryotic proteins marked as “verified experimentally”. This dataset, limited to the first 60 amino acids of each protein, was submitted to the CD-HIT program to obtain a non-redundant dataset with an identity cut-off at 30%. After this step, we obtained 1765 sequences with signal peptides in the *SPexp* dataset. One part of the *SPexp* dataset - 1000 sequences - and the totality of the 435 TM segments were divided at random into one training dataset called *SWPLearning* (305 TM, 700 SP) - Additional file 2 - and one test dataset called *SWPTest* (130 TM, 300 SP) – Additional file 3.

Our method is benchmarked using the SCAMPI datasets [14] and another derived from a recent extraction of TM protein segments from the PDBTM database [40] for which reduction to a non-redundant set was performed with an identity cut-off of 30% and completed by the remaining 765 sequences from the *SPexp* dataset. The resulting datasets were referred to as *ScampiHigh* – Additional file 4 - and *ScampiLow* –Additional file 5 - respectively for SCAMPI TM datasets with high- or low-resolution data, and *PDBTMSeg –* Additional file 6*-*. It is important to note that there is no redundancy between the *PDBTMSeg* dataset and the *SWPLearning* and *SWPTest* datasets. Finally several sequence selections from the ‘Benchmark of membrane helix predictions from sequences’ site [32] were used to evaluate PMIscale on PDB datasets.

### Local search algorithm for averaged values

Our algorithm, used to determine if a training segment is an SP segment or a TM segment, is based on a sliding-window approach. The value of a window is calculated as:

(1) $$\mathrm{Hi}=\frac{1}{n}\sum_{j=i}^{i+n-1}h\left(\mathrm{rj}\right)$$

where *i* is the position of the first residue within the sliding window, r is the residue at position j in the sequence, *n* is the length of the fixed window, and *h(rj)* is the PMI value. We define the PMI value of a sequence as the maximum value obtained when sliding the window along the sequence. If this value exceeds a threshold τfirst the sequence is considered as a TM segment. Otherwise, it is considered as a signal peptide.

PMI values are optimized by a local search method in order to obtain the best discrimination between SP and TM segments. Local search algorithms are modern heuristic methods designed for tackling hard optimization problems (see [41] for a review of these methods and their applications).

A local search algorithm starts with an initial candidate solution of the given search space and iteratively moves from the current solution to a neighboring solution that improves the function that must be optimized. At each step of the local search algorithm, all candidate neighbors of the current solution are evaluated. According to the steepest hill-climbing strategy, the best solution among the neighbors is chosen to replace the current solution, and the local search process is iterated from this new solution. The quality of a neighbor solution is assessed by an evaluation function based on the Area Under the ROC Curve (AUC) [22] with the ROCR package [42]. It estimates the ability of the solution to obtain a suitable discrimination between SP and TM segments.

A candidate solution is a set of 20 PMIscale values, one for each amino acid. The initial PMIscale values are set with the Kyte & Doolittle hydrophobic indexes. In a candidate solution, a PMIscale value is treated in three different ways to obtain a neighboring solution. It can be kept unchanged, and increased or decreased by a delta variation. Candidate neighbor solutions are obtained by combining these possible transformations of each PMI scale value.

Furthermore, we must keep in mind that we consider the localization information given by the SwissProt databank as approximate. To allow the movement of the window of maximum value along the sequence, significant valuable delta variations [+3, -3] are tested in the first iteration of the local search. These delta variations were gradually decreased during the following iterations. A systematic search including modifications of the 20 amino acid values at each step would be too time consuming. Therefore, to overcome this limitation, three groups of amino acids were defined. The local search process first deals with the first group, G1, and determines the optimal values for the amino acids of G1. It then searches the optimal values for the amino acids of the second group, G2, and finally deals with the third group, G3, in the same way. Results presented in this paper are obtained with the groups G1 = {F,L,I,V,Y,W}, G2 = {A,T,D,E,R,G,H} and G3 = {C,K,S,M,N,P,Q} which is the best grouping that has been tested. Nevertheless, we can note that several runs executed with several amino acid groupings gave slightly similar results. Learning performances vary also slightly according to a small variation of the length of the fixed window from 21 to 25 – the AUC decreases less than 6%. Nevertheless, the best performance is obtained with a fixed length equal to 23 amino acids.

### The localization of TM segments

To determine the localization of the TM segments, we developed a straightforward algorithm. A sliding window of fixed length (n = 23 residues, consistent with the learning dataset) is scanned across the protein sequence and a PMI value is calculated with Eq. (1) at each position along the sequence. The first window position that gives the PMI value above τfirst localizes the first TM segment. The iterative process continues to localize the other segments with a threshold τnext. Moreover, at least two amino acids separate two consecutive TM segments.

A threshold value equal to 2.7 equilibrates the confusion between signal peptides and signal anchors on the *SWPlearning* dataset – ie the number of SP predicted as signal anchors is roughly equal to the number of signal anchors predicted as SP. The minimal threshold τfirst required to predict the insertion of the first TM segment was extrapolated from this observation. Next, we evaluated τnext with the *PDBTMSeg* dataset. We chose to optimize the specificity rather than the sensitivity parameter, because our hypothesis is that some TM segments requires help to insert into the membrane and so, it is expected that some TM will be missed. The specificity value is set on the level of the best performing prediction methods – ie 0.96 – which leads to τnext = 2.1. All our benchmarks in this paper have been performed only with these two thresholds: τfirst = 2.7 and τnext = 2.1. Evolution of predictions according to the threshold τfirst and τnext are available in Additional file 1: Table S4.

### Availability of supporting data

Several data sets supporting the results of this article are included within the article and its additional files.

## Competing interests

The authors declare that they have no competing interests.

## Authors’ contributions

DT contributed to the conception and the development of the algorithms, manuscript preparation, and analysis of the results. SL contributed to the dataset preparation, conception and development of the algorithms. BD contributed to the conception of the algorithms, analysis of the results and manuscript preparation. EMR contributed to performance analysis, web development, analysis of the results and manuscript preparation. WBC contributed to performance analysis, analysis of the results and manuscript preparation. JKH contributed to the conception of the algorithms and manuscript preparation. All authors drafted the manuscript, revised it critically, read and approved the final version.

## Supplementary Material

Additional file 1: Table S1 Complementary assessments of the discrimination between SP and TM segments on the SWPTest dataset. **Table S2.** Evaluation of methods for the prediction of TMH localization and the prediction of membrane proteins (MP). **Table S3.** Influence of the length of the fixed-window on PMIscale predictions. **Table S4.** Influence of the τfirst and τnext parameters. Click here for file

Additional file 2 **The *SWPLearning* dataset.** Click here for file

Additional file 3 **The *SWPTest* dataset.** Click here for file

Additional file 4 **The *ScampiHigh* dataset.** Click here for file

Additional file 5 **The *ScampiLow* dataset.** Click here for file

Additional file 6 **The *PDBTMSeg* dataset.** Click here for file
