# Supplementary material for: In silico evaluation of the influence of the translocon on partitioning of membrane segments
Source: BMC Bioinformatics. 2014 May 21;15:156. doi: 10.1186/1471-2105-15-156 (PMC4035737; doi:10.1186/1471-2105-15-156)
Supplement: Additional file 1: Table S1 — Complementary assessments of the discrimination between SP and TM segments on the SWPTest dataset. Table S2. Evaluation of methods for the prediction of TMH localization and the prediction of membrane proteins (MP). Table S3. Influence of the length of the fixed-window on PMIscale predictions. Table S4. Influence of the τfirst and τnext parameters. [file 1471-2105-15-156-S1.pdf]

Supplementary Information

Table S1. Complementary assessments of the discrimination between SP and TM segments on the SWPTest dataset.

SignalP V4.1 and Phobius were both run with default parameters. "SP FN-rate" gives the percentage of SP that are not recognized as SP, and "TM FN-rate" gives the percentage of TM that are not recognized as TM segments.
Remark: The dataset used in this comparison may not be completely independent from the training datasets used in the Phobius and in the SignalP V4.1 methods.

	SP FN-rate %	TM FN-rate %	Accuracy
**SWPTest dataset**			
SignalP V4.1	9	3.1	0.93
Phobius	7.7	20.0	0.88
PMIscale	8.3	22.3	0.87

Table S2. Evaluation of methods for the prediction of the TMH localization and the prediction of membrane proteins (MP)

The benchmark contains 599 proteins with 133 membrane proteins - 101 TMH proteins, 2 proteins with only helices < 9 aa, 30 BB proteins - and 466 soluble proteins. This benchmark is referred as the TMH_1/2MH_OPM_BB_SOLB dataset in Rath *et al*. [28]. A predicted TMH is considered to be correct if it has an overlap of at least 5 residues with the reference protein. A membrane protein (MP) is considered as correctly predicted if at least one TMH or BB is predicted. As most of the programs are not trained to recognize BB membrane proteins or half-helices, the number of correctly TMH membrane proteins is also indicated in brackets.

Method	% of protein correctly predicted (all the TMH > 8 aa are correct)	Sensitivity	Specificity	Number MP (TMH) correctly predicted	Number soluble proteins correctly predicted	% of protein correctly assigned (MP or soluble)
Philius	93	90	96	96 (96)	462	93
Pro-TMHMM (in TOPCONS)	92	90	95	97 (97)	460	93
TOPCONSs	92	89	95	96 (96)	441	93
HMMTOP (in TOPCONS)	92	89	95	95 (95)	463	93
SCAMPI (TOPCONS single)	92	88	94	96 (96)	459	93
SCAMPI	91	88	94	96 (96)	459	93
SCAMPI sequence	91	88	94	96 (96)	459	93
Phobius	91	87	95	93 (93)	460	92
TMHMM2	91	86	96	92 (92)	464	93
SVMtop	91	86	95	87 (87)	463	92
TMMOD	91	85	96	91 (91)	461	92
S-TMHMM	91	85	95	90 (90)	462	92
PolyPhobius	90	91	92	98 (98)	448	91
MINNOU	90	89	91	95 (94)	452	91
MEMSAT	90	87	93	95 (95)	459	92
SVMtm	90	81	95	85 (85)	462	91
TOPCONS	89	91	92	97 (97)	441	90

SCAMPI-multi	89	90	92	96 (96)	441	90
OCTOPUS (in TOPCONS)	89	90	92	96 (96)	441	90
PHDThtm	89	82	93	0 (0)	466	78
SOSUI	88	82	93	91 (91)	455	91
DAS2002	88	82	92	96 (96)	446	90
**PMIscale**	**88**	**80**	**90**	**92 (91)**	**451**	**91**
PRED-TMR	86	83	89	98 (96)	436	89
DAS-TMfilter	86	81	91	94 (93)	445	90
HMMTOP2	85	90	84	99 (98)	416	86
OCTOPUS	84	94	83	99 (98)	399	83
HMM-TM	84	63	82	93 (88)	420	86
SPLIT4	82	75	87	94 (94)	427	87
MemBrain	81	94	79	102 (98)	390	82
waveTM	78	88	79	109 (97)	402	85
deltaG	75	89	75	107 (100)	365	79
TMAP	75	84	76	101 (95)	376	80
PHDhtm	72	77	70	104 (100)	364	78
DAS1997	71	82	67	104 (99)	345	75
VALPRED2	64	93	54	111 (97)	329	73
TMpred	64	86	64	120 (100)	325	74
VALPRED	63	76	61	110 (99)	314	71
TOPPRED2	61	85	62	118 (100)	301	70
DAS1997(strict)	37	89	39	126 (101)	173	50
PRODIV-TMHMM	32	94	43	130 (100)	123	42
KyteD (7,10)	22	84	25	132 (100)	96	38
OHM (7,10)	16	83	22	132 (101)	62	32
KyteD (11,10)	16	75	21	131 (101)	56	31
MEMSAT3	15	88	46	132 (100)	25	26
MEMSAT-SVM	12	91	46	132 (100)	2	22
OMH (11,10)	10	72	16	131 (101)	29	27
Eisen (7,10)	7	74	13	133 (101)	25	26
Eisen (11,10)	5	59	11	133 (101)	16	25
OHM (19,10)	2	51	12	133 (101)	0	22
KyteD (19,10)	1	58	16	133 (101)	0	22
Eisen (19,10)	1	41	9	133 (101)	0	22

Table S3. Influence of the length of the fixed-window on PMIscale predictions. The benchmark uses the TMH_1/2MH_OPM_BB_SOLB dataset described in Rath *et al*. [28].

Length of the window	% of protein correctly predicted (all the TMH are correct)	sensitivity	specificity
21	86	83	85
23	88	80	90
25	86	73	93

Table S4. Influence of the τfirst and τnext parameters. The benchmark uses the TMH_1/2MH_OPM_BB_SOLB dataset described in Rath *et al*. [28].The first number refers to the percentage of protein correctly predicted and the numbers in brackets refer to the sensitivity - the specificity of TM localization.

**τnext** / **τfirst**	1.6	2.0	2.1	2.2	2.3

2.5	84 (88-75)	84 (83-84)	85 (81-85)	85 (79-86)	84 (77-87)
2.6	86 (86-78)	86 (82-87)	87 (80-88)	87 (78-89)	86 (76-90)
2.7	87 (85-80)	87 (81-88)	88 (80-90)	87 (78-90)	87 (76-92)
2.8	87 (84-82)	87 (80-90)	88 (79-92)	88 (77-92)	88 (75-93)
2.9	87 (82-85)	88 (78-91)	88 (77-93)	88 (75-94)	88 (72-95)
